# A novel antimicrobial peptide screened by a *Bacillus subtilis* expression system, derived from *Larimichthys crocea* Ferritin H, exerting bactericidal and parasiticidal activities

**DOI:** 10.3389/fimmu.2023.1168517

**Published:** 2023-05-18

**Authors:** Meiling Chen, Nengfeng Lin, Xiande Liu, Xin Tang, Zhiyong Wang, Dongling Zhang

**Affiliations:** ^1^ Key Laboratory of Healthy Mariculture for the East China Sea, Ministry of Agriculture and Rural Affairs, Jimei University, Xiamen, China; ^2^ Institute of Biotechnology, Fujian Academy of Agricultural Sciences, Fuzhou, China

**Keywords:** antimicrobial peptide, *Bacillus subtilis*, antibacterial, antiparasite, Ferritin heavy subunit, *Larimichthys crocea.*

## Abstract

Antimicrobial peptides (AMPs) may be the most promising substitute for antibiotics due to their effective antimicrobial activities and multiple function mechanisms against pathogenic microorganisms. In this study, a novel AMP containing 51 amino acids, named Lc1687, was screened from the large yellow croaker (*Larimichthys crocea*) *via* a *B. subtilis* system. Bioinformatics and circular dichroism (CD) analyses showed that Lc1687 is a novel anionic amphiphilic α-helical peptide, which was derived from the C-terminal of a Ferritin heavy subunit. The recombinant Lc1687 (named rLc1687) purified from *Escherichia coli* exhibited strong activities against Gram-positive (Gram+) bacterium *Staphylococcus aureus*, Gram-negative (Gram-) bacteria *Vibrio vulnificus, V. parahaemolyticus*, and *Scuticociliatida*. Scanning electron microscope (SEM) and transmission electron microscopy (TEM) revealed the possible function mechanisms of this peptide, which is to target and disrupt the bacterial cell membranes, including pore-forming, loss of fimbriae, and cytoplasm overflow, whereas gel retardation assay revealed that peptide Lc1687 cannot bind bacterial DNA. The peptide stability analysis showed that rLc1687 acts as a stable antimicrobial agent against Gram+ and Gram- bacteria at temperatures ranging from 25 to 100°C, pH 3-12, and UV radiation time ranging from 15 to 60 min. A hemolytic activity assay confirmed that this peptide may serve as a potential source for clinical medicine development. Taken together, Lc1687 is a novel AMP as it is a firstly confirmed Ferritin fragment with antimicrobial activity. It is also a promising agent for the development of peptide-based antibacterial and anti-parasitic therapy.

## 1 Introduction

The discovery and use of antibiotics is one of the most important breakthroughs in medical history. However, the widespread use and misuse of antibiotics have led to the continuous acquisition of new genetic traits and resistance genes by bacteria. Furthermore, drug residues in food and the environment pose a great threat to human public health. These problems have become a major obstacle to the healthy development of aquaculture. In various substrates of mariculture farms, antibiotics resistance genes have extremely high abundances and pose potential ecological health risks (1, 2). Additionally, recently, the World Health Organization (WHO) highlighted that most antibiotic candidates are merely modifications of existing molecules and do not target drug-resistant Gram-negative (Gram-) bacteria. Nevertheless, infectious diseases in aquaculture are primarily caused by multi-resistant Gram- bacteria, and the water environment in which fish live is more conducive to the survival of bacteria and other microorganisms. Accordingly, the search for alternatives to synthetic antibiotics in aquaculture has become essential.

Antimicrobial peptide (AMP), as a native host defense peptide, is widely distributed in animals, plants, and microorganisms. It is routinely described as a class of small-molecular peptides (within 100 amino acids in length) with significant activities against bacteria, parasites, viruses, fungi, tumor cells, and inflammation (3). Most AMPs are cationic peptides, containing 2 to 13 positive charges, with a high proportion of hydrophobic residues (typically 50%) (4). These features allow AMPs to interact with negatively charged bacterial surfaces *via* electrostatic attraction and hydrophobic insertion causing membrane disorganization by inducing toroidal pore (i.e., wormhole), barrel-stave, and carpet model phenomena and, eventually, cell death (5). Besides these, some AMPs can penetrate the cell membrane to reach the interior of the cell, where they block critical cell processes, such as disturbing protein/nucleic acids synthesis, protein/enzyme activity, cell division, protein proper folding, etc. (6). In addition to killing bacteria directly, AMPs affect the host response to infection in multiple ways, including the modulation of chemokine and cytokine production, angiogenesis, endotoxin neutralization, and wound healing (7, 8). Furthermore, AMPs can affect the maintenance of the integrity of the intestinal barrier by stimulating mucus synthesis and promoting the production of tight junction proteins and the repair of the endothelium (9, 10). Interestingly, accumulating evidence shows the synergistic effects between AMPs or between AMPs and antibiotics, and these combinations can significantly reduce bacterial resistance (11–13). Owing to these favorable characteristics with infrequent drug resistance, broad-spectrum antimicrobial activities, rapid killing effects, and high capacity for synergies, AMPs have been considered attractive candidates for alternative antibiotics.

AMPs can be rapid screened from the natural source through various chromatography and high throughput sequence methods (14, 15). However, these methods are relatively complicated, and the predictions are not accurate enough. *Bacillus subtilis* is generally recognized as a safe (GRAS) food-grade microbial system. Due to its clear inherited background, simple and diverse genetic manipulation systems, high growth rate, short fermentation time, strong secretory ability, and accumulative fermentation experience, *B. subtilis* has been widely used as a cell factory for the microbial production of chemicals, enzymes, and antimicrobial materials in industry, agriculture, and medicine.

The large yellow croaker (*Larimichthys crocea or L. crocea*) is an important marine economic fish and is one of the traditional “four marine products” in China. *L. crocea* is extremely susceptible to bacteria and parasites, which lead to enormous economic damage (16, 17). Currently, there are numerous problems surrounding the culture of *L. crocea*, including bacterial multi-resistance, the absence of effective drugs against parasites, food drug residues, and environmental contamination of antibiotics. Therefore, there is an urgent need to develop natural antimicrobial agents.

In the current study, a novel AMP was identified from *L. crocea* using a *B. subtilis* expression system, and further studies were performed to explore their antibacterial and anti-parasitic activities. To determine the basis of the host–pathogen interaction, the mechanism of action of AMP was investigated, and the stabilities and safety of this peptide were also estimated.

## 2 Materials and methods

### 2.1 Pathogen cultures and fish tissue collection

*Staphylococcus aureus, Pseudomonas plecoglossicida, Vibrio vulnificus*, and *V. parahaemolyticus* were preserved in our laboratory. *B. subtilis* SCK46 was presented by Professor Wubei Dong from Huazhong Agricultural University in Wuhan, China. All bacteria were cultured on Luria-Bertani (LB) media at 37 °C or 28 °C. *Scuticociliatida* was isolated from *L. crocea* by our team and maintained in our laboratory. Specifically, in mid-April 2019, the infected fish was obtained from Fuding City, Fujian Province, China. The fish was dissected, and the gills were put into a plate full of seawater. *Scuticociliatida* swam in seawater and were then drawn into a 6-well plate. The parasites were cultured at 16 °C and fed *Escherichia coli* once every day.

Health *L. crocea* (~10 g) was obtained from a Guanjingyang Company Limited in Ningde, Fujian province, China. The fish was acclimatized in the filtered and aerated seawater (25 °C) and fed a commercial feed twice daily for two weeks before conducting the experiments. The immersion method was used in the challenge experiment. The *Pseudomonas plecoglossicida* was cultured for 18 hours before challenge, and the fish was exposed to bacteria at a concentration of 1×10^6^ CFU/mL for 3 hours. Samples (liver, spleen, and kidney) were collected at different time points (24h, 48h, 72h) and then immediately frozen in liquid nitrogen, and saved at -80 °C.

### 2.2 *L. crocea* cDNA library construction

Total RNA was extracted with TransZol™ Up Plus RNA Kit (Invitrogen, USA). The obtained mRNA was purified using PolyATtract^®^ mRNA Isolation Systems (Promega, Madison, WI, USA). The quality of total RNA and purified mRNA was detected by 1% agarose gel electrophoresis. cDNA library construction refers to Abbas’ method (18). Briefly, the Double-stranded cDNA was prepared with a specific oligo(dT) primer (containing an Xba I cleavage site). Three pairs of primers containing Nde I cleavage site adaptor were added to the cDNA library. The library and a pBE-S vector were then digested with Xba I and Nde I restriction enzyme (TaKaRa, Dalian, China), followed by their ligation by T4 ligase. Finally, the ligation products were transformed into *B. subtilis* SCK6 cells and incubated at 37 °C overnight. Individual colonies were picked, colony PCR was performed using pBE-S-F and pBE-S-R primers (**Table S1**) to confirm the cDNA library quality, and then colonies were saved at −80 °C.

### 2.3 Candidate genes screening

Transformants from the cDNA library were plated on LB plates containing kanamycin (10 mg/L) and incubated at 37 °C to observe the phenotype. The strains with lethal phenotype would be validated through three repeat bacterial spotting tests, and then the strains with stable lethal phenotype were sequenced.

### 2.4 Crude protein extraction

We only extracted the extracellular protein because *B. subtilis* has a high capacity to secrete protein directly into the extracellular medium. The strains with stable lethal phenotype were fermented at 180 r/min, at 37 °C for 72 h. The supernatant was collected by centrifugation at 10,000 r/min, 4 °C for 30 min. Saturated ammonium sulfate solution at the final concentration of 50%-60% was slowly added into the supernatant and stirred continuously on ice for at least 20 min. The solution was then maintained at 4 °C for overnight. The next day, the separated protein was centrifuged using the aforementioned conditions-. The collected precipitation was dissolved in 25 mM PBS (pH 7.0) and dialyzed in the same PBS buffer for 24 h at 4 °C. The insoluble debris was discarded by centrifugation.

### 2.5 Antibacterial activity analysis of the extracellular protein

Antibacterial activity assay of the extracellular protein was performed using disk diffusion. Indicator bacteria *S. aureus, V. vulnificus*, and *V. parahaemolyticus* (1×10^8^ cfu/mL) were mixed with LB media and poured over previously prepared LB plates. Then, 4 mm diameter filter paper disks were placed on the agar plates and 20 µl extracellular proteins (1000 µg/mL) were added to each filter paper. Hereafter, the plates were incubated for 12 h at the different temperatures required for the different indicator bacteria. Antibacterial activity was confirmed by measuring the diameters of inhibition zones. The extracellular protein of null vector pBE-S was set as the control.

### 2.6 Bioinformatics analysis and circular dichroism spectrophotometry

Homology analysis of Lc1687 was performed using the BLAST program at NCBI (http://www.ncbi.nlm.nih.gov/blast). The molecular weight (MW) and isoelectric point (pI) of protein are predicted on the EXPASY server (http://www.expasy.org/). A three-dimensional protein model was constructed using Vector NTI Suite 8 and Phyre2 (http://www.sbg.bio.ic.ac.uk/phyre2, 30 September 2019). The helical wheel analysis was performed with Heliquest. The bioinformatics results were corroborated by the findings of circular dichroism (CD).

The circular dichroism analysis was carried out using a spectropolarimeter (Chirascan V100, Applied Photophysics Ltd). The peptide was dissolved in 0.2 M PBS (pH 7.3) to a final concentration of 1.0 mg/mL, and PBS served as the control. The samples were put into 1 mm path length quartz cuvettes and the data were recorded from 190 to 260 nm. The scanning speed of the CD spectrum was 100 nm/min, with data recorded at 1.0 nm intervals. The circular dichroic absorption value was calculated using the following equation: [ϴ]= mdeg×M/(l0×c×nr). Where mdeg is the CD value, M is the molar mass of the peptide, c is the concentration of the peptide, and nr is the number of amino acid residues.

### 2.7 Expression and purification of antimicrobial peptide

Antimicrobial peptide Lc1687 was expressed using a pET-32a vector. Primers Lc1687-F/R (**Table S1**) with restriction sites were used to amplify the *Lc1687* gene. The target PCR fragment was inserted into the expression vector pET-32a with TRX-His-tag after being digested with EcoRI and XhoI restriction enzymes (TaKaRa, Dalian, China). The recombinant plasmid (pET-32a-Lc1687) and the parent vector pET-32a (negative control) were both transformed into *E. coli* BL21 (DE3) and expressed with IPTG induction at 20 °C for 16 h. The bacteria were harvested by centrifugation at 7,000 r/min for 20 min and resuspended in buffer A (20 mM imidazole, 10 mM Na_2_HPO_4_, 140 mM NaCl, 1.8 mM KH_2_PO_4_, 2.7 mM KCl, pH 7.4) for sonication. Afterward, the supernatant was collected by centrifugation at 12,000 g for 20 min and loaded onto a His-tag column. The His-tag column was equilibrated with buffer A, followed by washing with buffer B (40 mM imidazole, pH 7.4) to remove non-target proteins, and, finally, the target protein was collected with elution buffer (500 mM imidazole, pH 7.4). The purified protein was analyzed by 12% SDS-PAGE and dialyzed with PBS buffer (0.01M, pH 7.4) for desalination.

### 2.8 Antibacterial activity analysis and minimum inhibitory concentration determination of recombinant Lc1687 (rLc1687)

Antibacterial activity assay of the recombinant protein was performed using Oxford cup diffusion. The method was similar to the previously described disk diffusion, except that the filter paper disk was replaced with an Oxford cup, the protein 50 µl (500 µg/mL) was added into the cup, and the empty vector protein was set as the control. In addition, the protein was diluted into 1000, 500, 250, 125, 62.5, 31.25, and 15.63 µg/mL with PBS (0.01 mol/L, pH 7.4) to determine the MIC. The three inhibition zones on the same solid plate are for three experimental replicates.

### 2.9 Scanning electron microscopy

Bacterial cell surface morphology was observed by SEM. Indicator bacteria (1.0×10^8^ cfu/mL) were incubated with the same volume of antimicrobial peptide rLc1687 (500 µg/mL) at the different temperatures required for the bacteria for 2 h. The empty vector protein was substitute for rLc1687 as the control. The samples were subsequently fixed with 2.5% glutaraldehyde aqueous solution for 3 h at 4 °C and dehydrated in a series of ethanol solutions (50%, 70%, 90%, 95%, and 100%). Finally, the samples were lyophilized, gold coated, and observed using a scanning electron microscope (Hitachi S-4800, Japan).

### 2.10 Transmission electron microscopy

The ultrastructural changes in bacteria induced by antimicrobial peptide rLc1687 were examined by TEM. Indicator bacteria (1.0 ×10^8^ CFU/mL) were incubated with equal volume antimicrobial peptide rLc1687 (500 µg/mL) at 28 or 37 °C for 1.5-2 h. The bacteria added to empty vector protein served as the controls. The samples were dropped onto a cropper mesh, followed by 2.0% phosphotungstic acid negative staining for 60 sec before being imaged on an H-7650 transmission electron microscope (Hitachi, Japan).

### 2.11 Gel retardation assay

To explore whether the AMP destroys nucleic acids of pathogenic bacteria, a gel retardation assay was performed to analyze bacterial DNA integrity. Bacterial genomes were extracted by FastPure^®^ Bacteria DNA Isolation Mini Kit (Vazyme, Nanjing, China). AMP rLc1687 was incubated with the bacterial genome in various proportions (4:1, 3:2, 2:3, and 1:4) at room temperature for 1 h, following the DNA integrity check using 1% gel electrophoresis. The bacterial genome was used as a blank control.

### 2.12 Anti-parasitic activity analysis of rLc1687

The isolated *Scuticociliatida* was cultured in a petri dish at 16 °C with sterilized seawater. On the third to fourth day, when the ciliates adapted to the culture and were able to expand steadily, they were used in the following experiment. Approximately 300 ciliates were added into a 48-well plate (a total volume of 1.5 mL) containing a final concentration of 500 µg/mL rLc1687 and then incubated at 16 °C. The ciliates were observed under an ordinary light microscope at 15 min, 30 min, 1h, and 2 h. The protein purified from the null vector was substitute for rLc1687 as the control.

### 2.13 Stability assay of rLc1687

To explore whether the antimicrobial activity of rLc1687 is affected by environmental factors, rLc1687 was treated with gradient temperature, pH, and UV irradiation time, then its antibacterial activity was detected using the Oxford cup diffusion method. The work concentration of rLc1687 is 1000 µg/mL. For the thermal stability test, the peptide was heated at 25, 50, 75, and 100 °C for 30 min before use. For the pH assay, rLc1687 solution was adjusted with HCL and NaOH to pH 3, 5, 7, 11, and 12. For UV irradiation, the peptide was irradiated for 15, 30, 45, and 60 min.

### 2.14 Hemolytic activity assay of rLc1687

The hemolytic activity of rLc1687 was assessed according to the previously described method with slight modifications (19). Briefly, fresh blood was taken from the *L. crocea* tail vein and centrifuged at 3,000 r/min for 5 min to collect the erythrocytes then washed three times with a TBS buffer (0.20 M, pH 7.2) and resuspended in the same solution (1.50% v/v). The erythrocyte suspension was mixed with different concentrations of protein solutions (0, 15.625, 31.25, 62.5, 125, 250, 500, 1000 µg/mL) in equal volumes. TBS buffer was used as a negative control (no hemolysis), and double-distilled water was used as a positive control (complete hemolysis). The hemolytic activity percentage was calculated using the following equation: hemolysis (%) = [(test group A_520_ - negative control group A_520_)/(positive control group A_520_ - negative control group A_520_)] ×100%.

### 2.15 Statistical analysis

All experiments were performed with at least three replicates. Data were expressed as the mean ± standard error of the mean (SEM). Statistical analysis was performed by a one-way ANOVA, LSD multiple comparison test, and independent sample t-test using SPSS 20.0. *P*<0.05* and *P*<0.01** indicated a statistical significance between groups.

## 3 Results

### 3.1 Candidate gene from an *L. crocea* cDNA library exhibited antimicrobial potential

**Figure 1:**
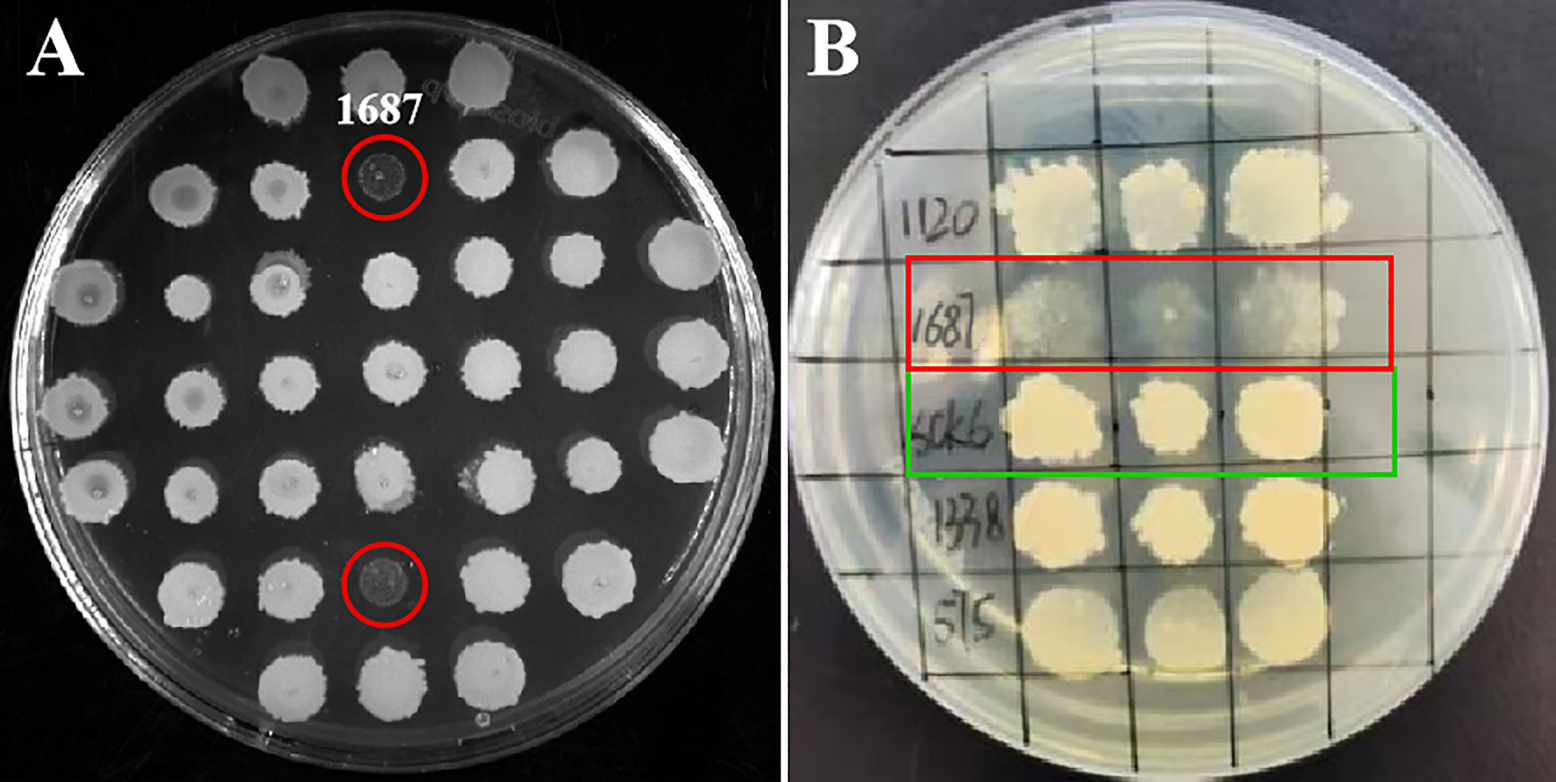
Screening antimicrobial gene from *L. crocea* cDNA library. The engineered *B. subtilis* strain 1687 (red marks) and SCK6 control (green mark) were separately spotted onto LB plates and incubated at 37 °C for 72 (h) **(A)** Initial screening of antibacterial genes; **(B)** Repeat screening of antibacterial genes (*n*=3).

A high-quality *L. crocea* cDNA library was constructed (**Figure S1**). A total of 3,086 clones from the cDNA library were spotted on LB solid media. During initial screening, strain 1687 exhibited cell lysis at 72 h post-incubation at 37 °C (**Figure 1A**). Three repeat tests for the bacterial spotting plate confirmed this result (**Figure 1B**). These investigations suggested that the protein encoded from the inserted fragment in the 1687 strain has the potential to inhibit or kill microorganisms.

### 3.2 The extracellular protein in the 1687 strain exhibited antibacterial activity

**Figure 2:**
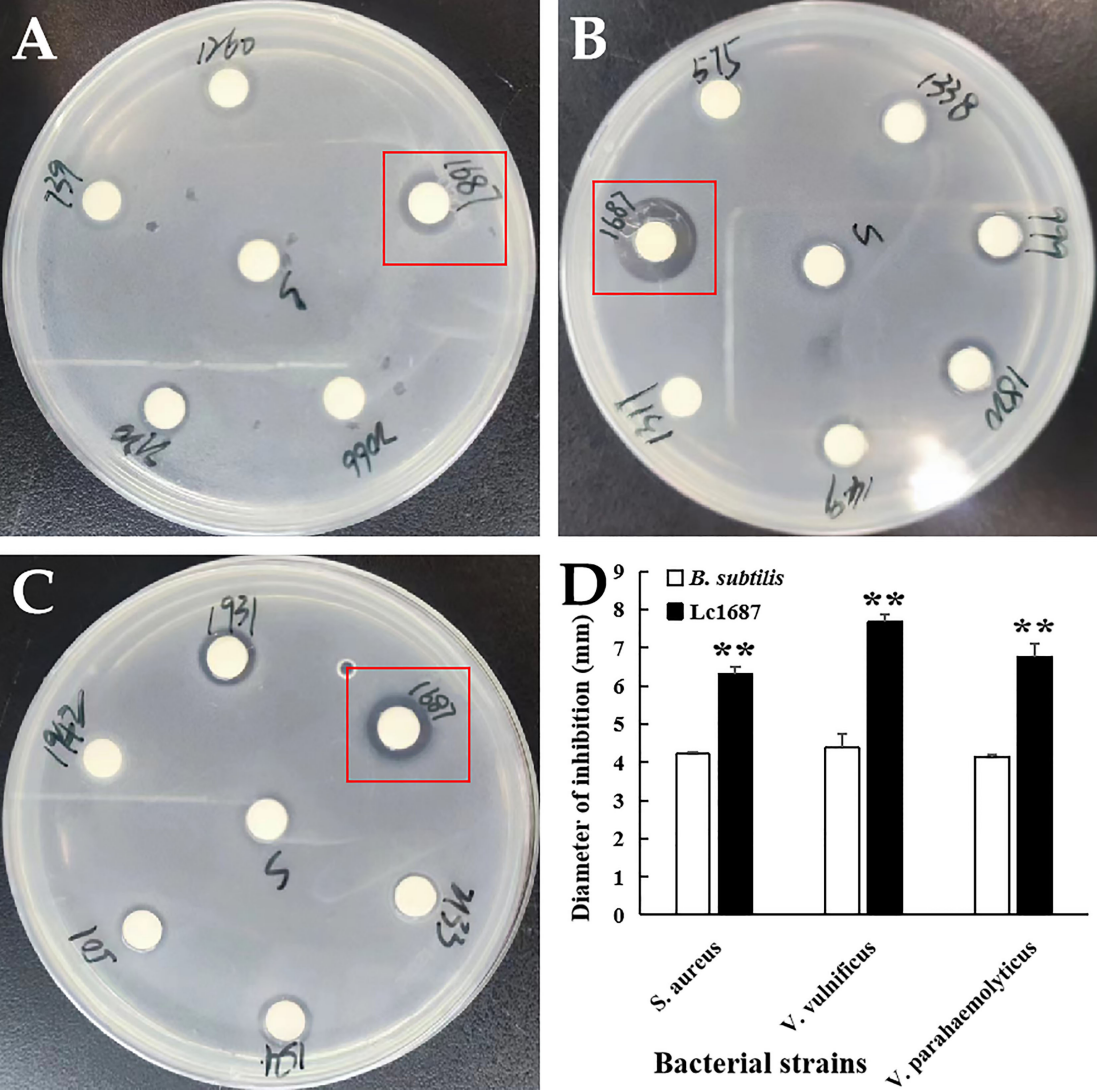
Antibacterial activity of extracellular protein Lc1687. **(A)** *S. aureus*; **(B)** *V. vulnificus;* **(C)** *V. parahaemolyticus;* **(D*)*** Diameter of inhibition zone. The meaning of the symbol ** is extremely significant.

Extracellular protein of the 1687 strain was extracted using the ammonium sulfate precipitation method, and its antibacterial activity was further detected using the disk diffusion method. The results revealed that the 1687 protein (named Lc1687) exhibited more significant antibacterial activity against Gram+ bacterium *S. aureus* as well as Gram- bacteria *V. vulnificus*, and *V. parahaemolyticus* compared to the control *B. subtilis* SCK6 (**Figure 2**). Among these, the strongest antibacterial activity exhibited by Lc1687 was against *V. vulnificus*. The results again confirmed that the *B. subtilis* 1687 strain harboring protein possesses the ability to restrict the growth of Gram+ and Gram- bacteria.

### 3.3 Lc1687 is a novel anionic amphipathic α-helical antimicrobial peptide derived from a Ferritin heavy subunit

**Figure 3:**
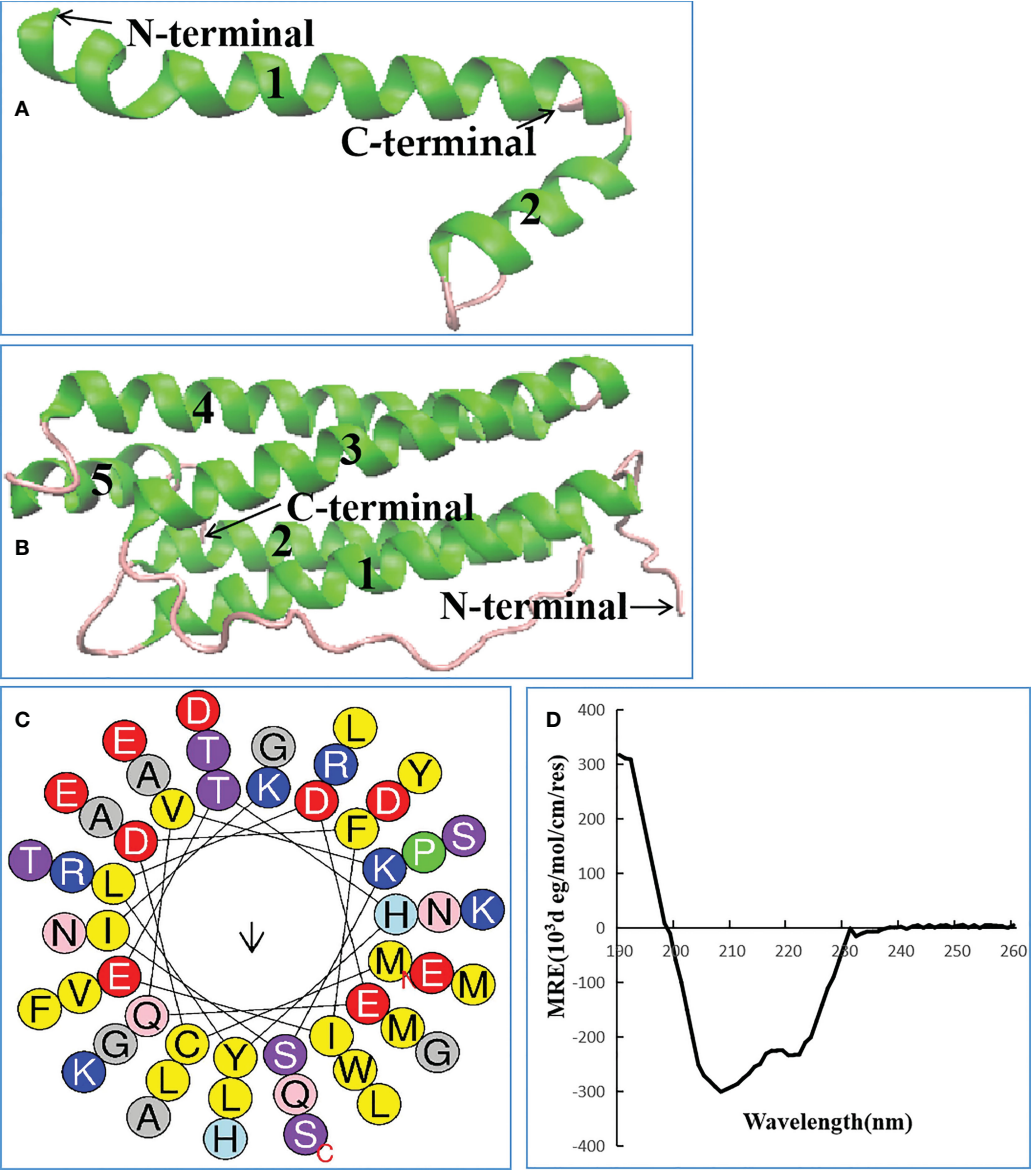
Biological characteristics of Lc1687. **(A)** Antibacterial peptide Lc1687, **(B)** *L. crocea* Ferritin (H) The numbers 1-5 represent α helical structure marked with green, and purple indicates random coil; **(C)** Helical wheel diagram. The black arrows indicate the direction of the hydrophobic moment, different kinds of residues are presented in different colors, hydrophobic residues are shown in yellow, and blue circles represent cationic residues; **(D)** Circular dichroism pattern at 25°C.

Lc1687 (CDFIETHYLDEQVKSIKELADWVTNLRRMGAPQNGMAEYLFD-KHTLGKESS) is a C-terminal fragment of a Ferritin heavy subunit (Ferritin H). The tertiary structure analysis showed that Lc1687 contains long and short α-helices, whereas the whole Ferritin H in *L. crocea* has five α-helical structures (**Figures 3A, B**). A helical wheel analysis implied that Lc1687 is an anionic antimicrobial peptide with a total net charge of -3 at neutral pH 7.4. Moreover, Lc1687 exhibits good amphipathic properties with a hydrophobicity of 0.311 and a hydrophobicity moment of 0.217. The hydrophobic face is composed of YLCL (Tyr, Leu, Cys, Tyr), whereas the charged residues are on the hydrophilic side (**Figure 3C**). The calculated molecular weight (MW) of Lc1687 is 6,047.87 Da with a theoretic isoelectric point (pI) of 5.11. Additionally, circular dichroism analysis revealed that peptide Lc1687 exhibited one positive absorption peak at 190 nm and two negative absorption peaks at around 208 nm and 220 nm in PBS solution, which is a typical α-helical structural characteristic of circular dichroism (**Figure 3D**), and the results are consistent with foregoing structure prediction (**Figure 3A**). This peptide is considered to be a novel antibacterial peptide because no previous reports have revealed which Ferritin fragment has the ability to restrict bacterial growth. And it had no homology with any peptide in the antimicrobial peptide database (APD). The series analysis showed that Lc1687 is a novel anionic amphipathic α-helical antimicrobial peptide derived from Ferritin H.

### 3.4 Capture and purification of recombinant protein

**Figure 4:**
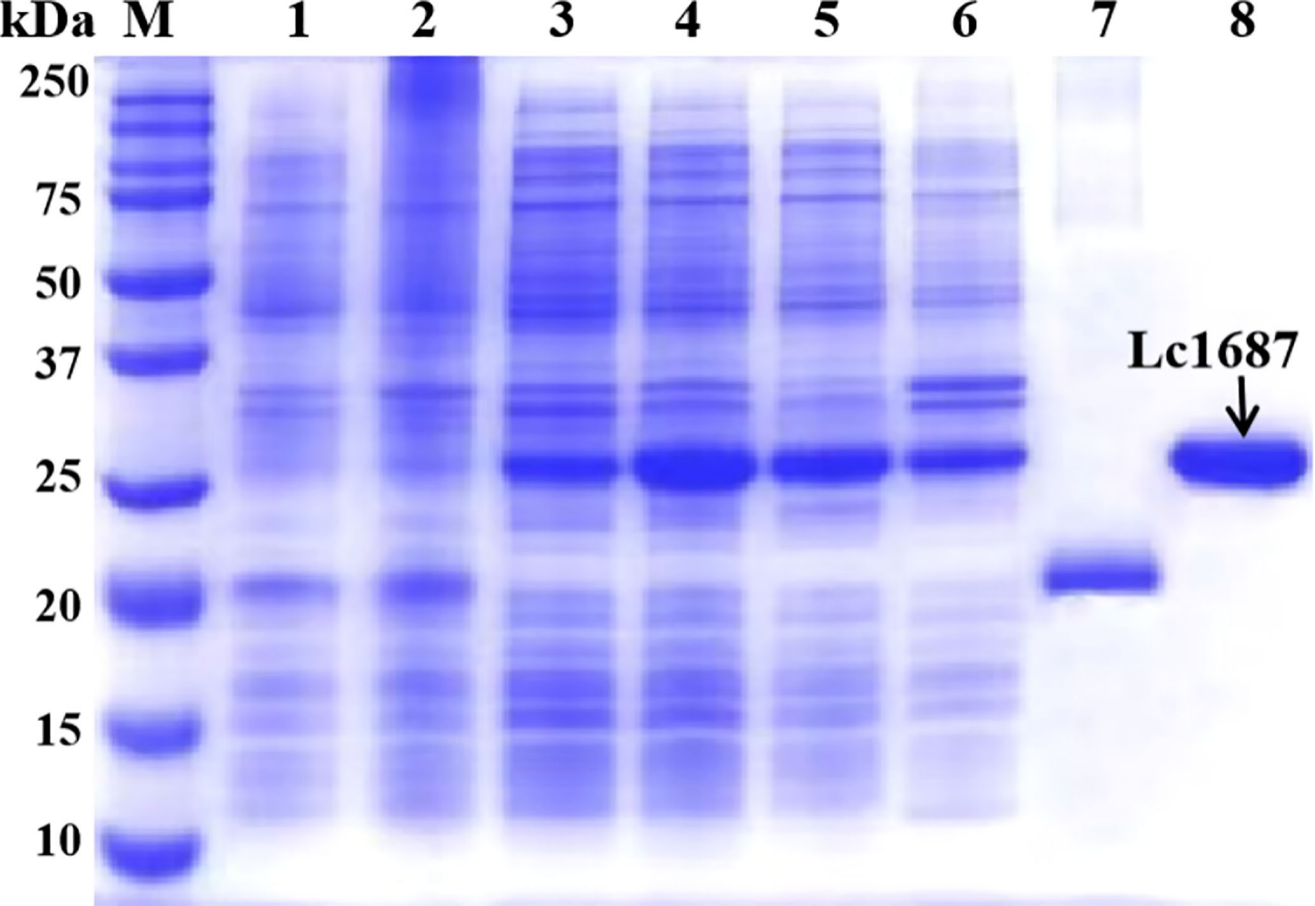
Expression and purification of recombinant Lc1687. Lane M, protein marker; 1: Non-induced empty vector pET-32a; 2: Induced empty vector pET-32a; 3: Non-induced pET-32a-Lc1687; 4: Induced pET-32a-Lc1687; 5: pET-32a-Lc1687 supernatant; 6: pET-32a-Lc1687 precipitate; 7: Purified Trx-His-tag; 8: Purified rLc1687.

The parent vector pET-32a and the plasmid pET-32a-Lc1687 were transformed into *E. coli* BL21 (DE3). After IPTG induction, purification, and SDS-PAGE analysis, one approximate 20 kDa (Trx tag + S tag + 6 × His tag) band was visualized (**Figure 4**, lane 7), and the recombinant Lc1687 (named as rLc1687) exhibited a distinct band at ~26 kDa (**Figure 4**, lane 4/5/8), corresponding to the prediction for Lc1687 6.05 kDa plus a 20 kDa of TRX-S-His-tag. The fusion protein mainly appeared in solution form (**Figure 4**-lane 5). The results indicated that rLc1687 was successfully expressed in a prokaryotic system.

### 3.5 rLc1687 possesses antibacterial activity in a low dose

**Figure 5:**
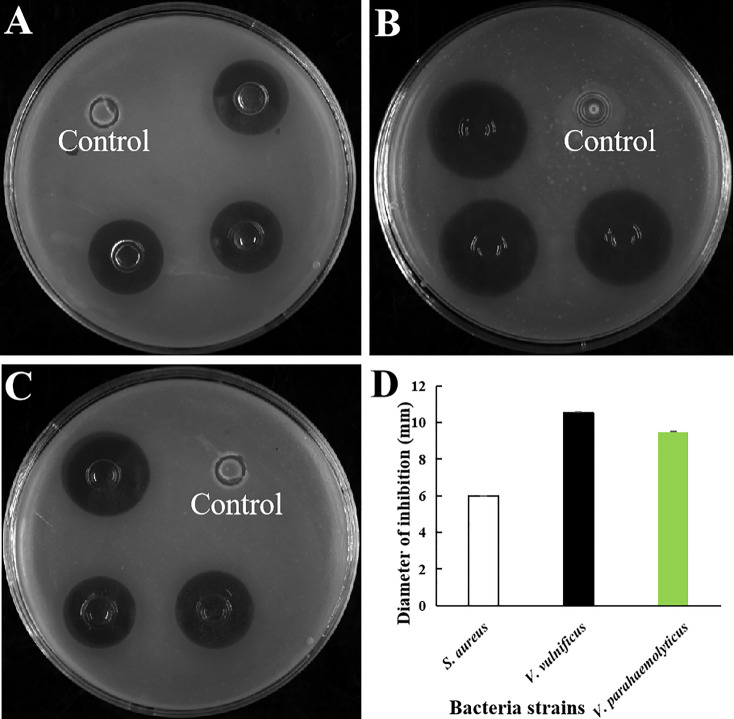
Antibacterial activity of rLc1687. **(A)** *S. aureus*; **(B)** *V. vulnificus*; **(C)** *V. parahaemolyticus*; **(D)** Diameters of inhibition zones. The empty vector protein was substitute for rLc1687 as the control. The three inhibition zones on the same solid plate served as three experimental replicates.

The antibacterial activity of purified rLc1687 was checked using the Oxford cup diffusion method. The results revealed that rLc1687 displayed significant levels of inhibition against *S. aureus*, *V. vulnificus*, and *V. parahaemolyticus* (**Figure 5**). Moreover, once again, the strongest antibacterial activity exhibited by peptide Lc1687 was confirmed to be against *V. vulnificus*, of the three indicated bacteria. The control empty vector protein exhibited no antibacterial activity against the three indicator bacteria.

**Figure 6:**
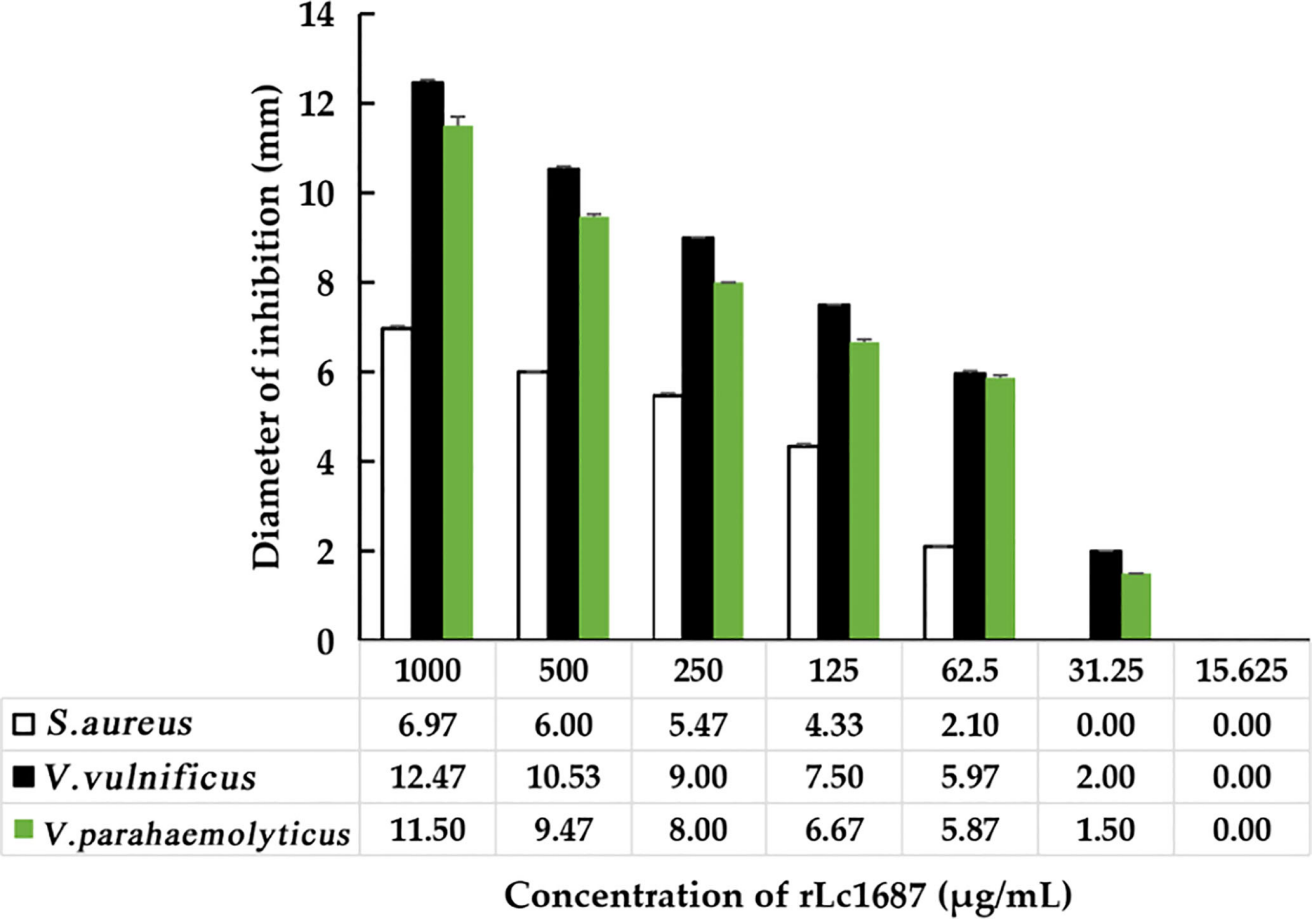
MIC determination of rLc1687 antibacterial activity against three indicator bacteria.

A serial dilution of rLc1687 was used to measure MIC with the Oxford cup diffusion method. As shown in **Figure 6**, with the decrease in rLc1687 concentration, the inhibition zone diameters of various indicator bacteria also gradually decrease. The diameter of the inhibition zone against *S. aureus* was 2.10 mm at an rLc1687 concentration of 62.5 µg/mL, and the diameters against *V. vulnificus* and *V. parahaemolyticus* were, separately, 2.0 *mm* and 1.50 mm at an rLc1687 concentration of 31.25 µg/mL. No activity was observed at lower protein concentrations. In conclusion, the MICs of rLc1687 against the three indicator bacteria were 31.25-62.5, 15.625-31.25, and 15.625-31.25 µg/mL, respectively.

### 3.6 Antibacterial mechanisms analysis of rLc1687

**Figure 7:**
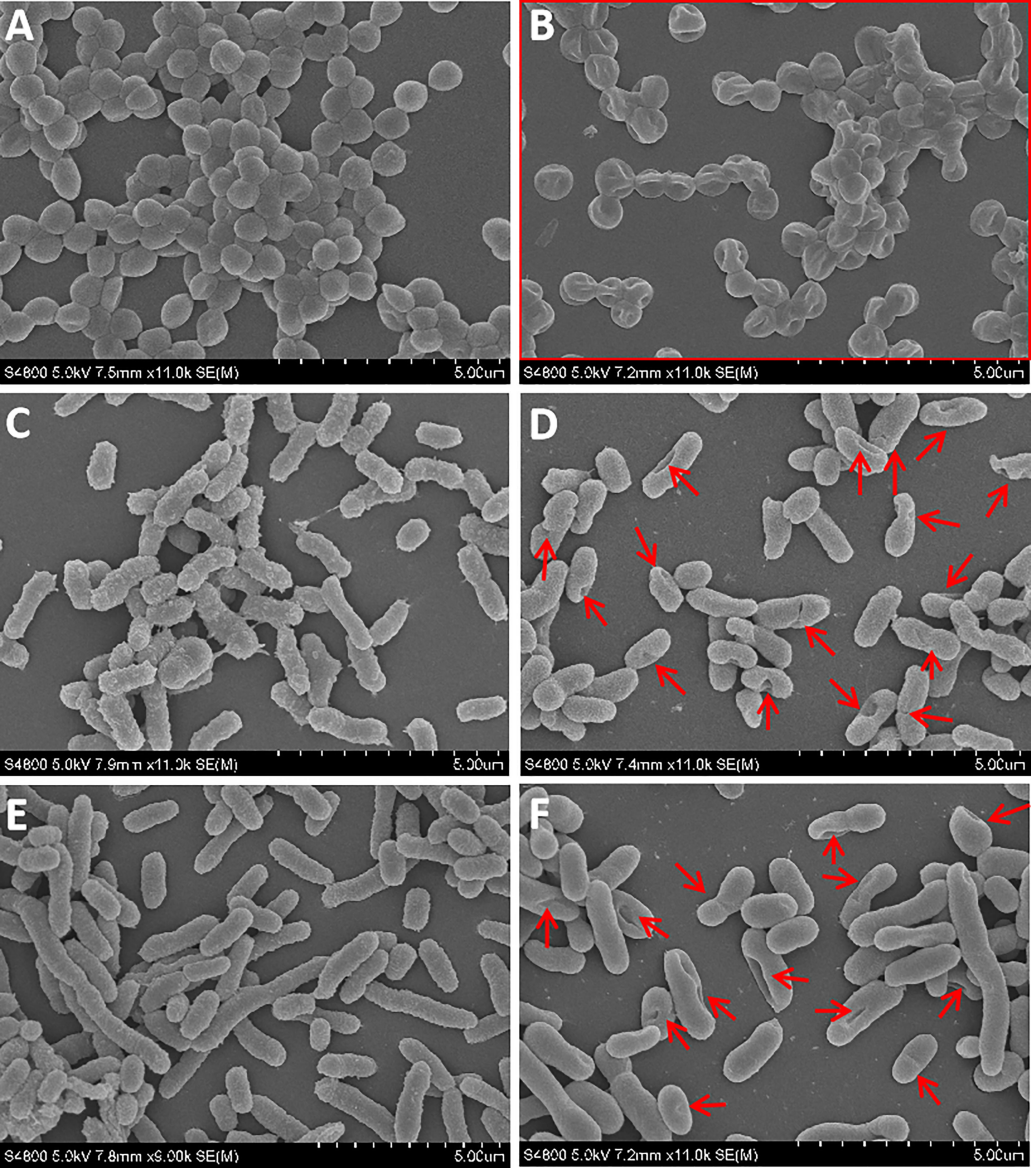
SEM observation on the damaged bacteria. **(A, C, E)** *S. aureus*, *V. vulnificus*, and *V. parahaemolyticus* blank control; **(B, D, F)** *S. aureus*, *V. vulnificus*, and *V. parahaemolyticus* were incubated with rLc1687, respectively; The rectangles and red arrows represent damaged bacteria. The empty vector protein was substitute for rLc1687 as a control.

**Figure 8:**
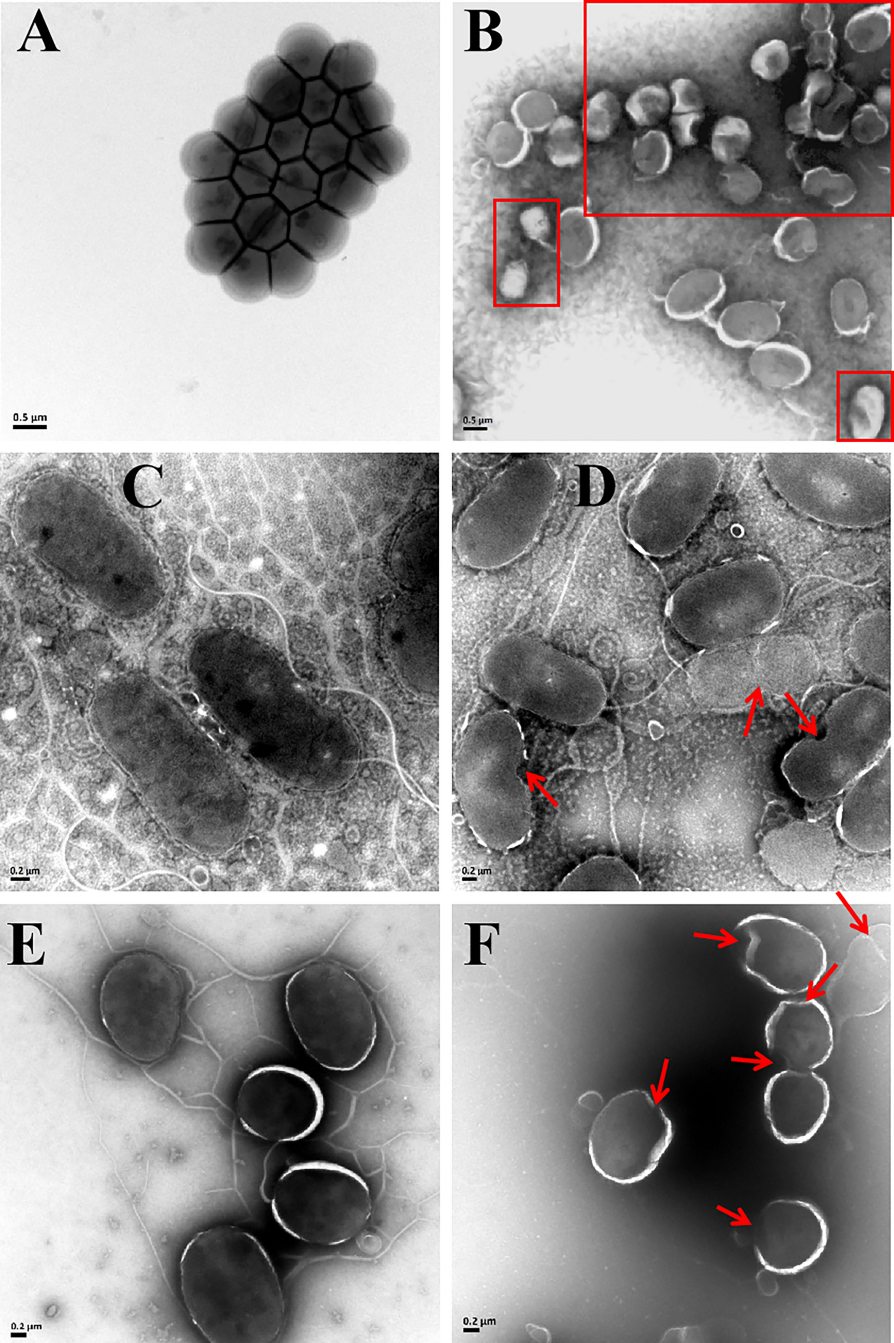
TEM images of *S. aureus* **(A, B)**, *V. vulnificus* **(C, D)**, and *V. parahaemolyticus* **(E, F)** under the empty vector protein **(A, C, E)** and rLc1687 treatments **(B, D, F)**, respectively. The rectangles and red arrows represent damaged bacteria.

The bactericidal mechanism of AMPs is commonly through the disruption of cell membranes or through targeting certain intracellular components, such as DNA. To investigate the possible mechanisms for the bactericidal function of rLc1687, firstly, we employed the SEM method to visualize bacterial surface integrity (**Figure 7**). The SEM results revealed that the three indicator bacteria showed noticeable membrane pore formation and cytoplasm outflow after treatment with rLc1687, but the three bacterial morphologies were different. *S. aureus* shrinks exceptionally (**Figures 7A, B**), and the cells became deformed and stuck together, whereas two vibrio strain (*V. vulnificus* and *V. parahaemolyticus*) surfaces became smoother, compared to the controls, due to the loss of pilis (**Figures 7C–E**). The bacterial ultrastructure changes were further examined by TEM; the control group bacteria had evenly distributed intact cell membranes, whereas the test group bacteria had drastic changes (**Figure 8**). Deformation and disruption of cell walls and cell membranes, as well as cytoplasm outflow, were observed in the test group. Finally, we wondered whether rLc1687 kills the three bacteria by targeting intracellular molecules. The results show that rLc1687 cannot bind directly to the genomic DNA of the three indicator bacteria (**Figure S2**). Collectively, rLc1687 kills bacteria by disrupting the membrane integrity and leading to cytoplasm overflow for both Gram+ and Gram- bacteria, without binding bacterial DNA.

### 3.7 rLc1687 exhibits potent killing *Scuticociliatida* directly

**Figure 9:**
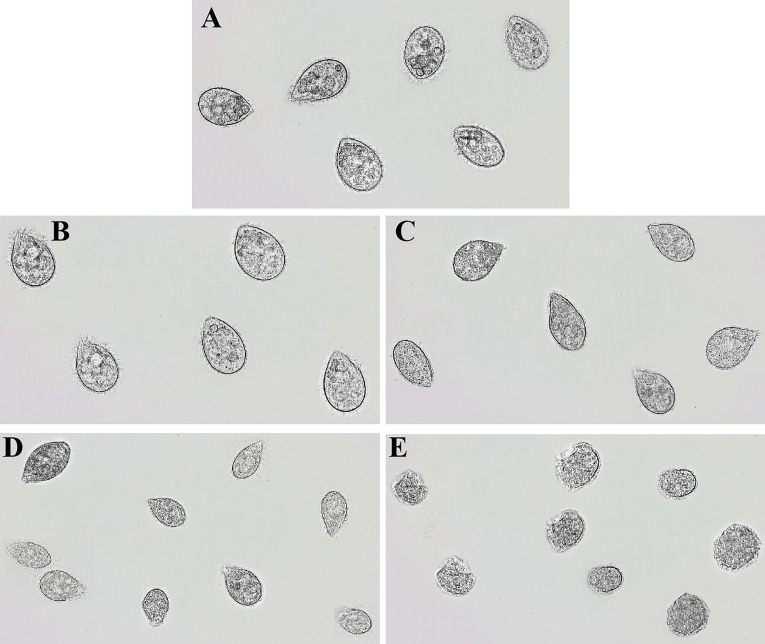
Anti-*Scuticociliatida* activity of rLc1687. The ciliates were cultured without **(A)** and with **(B–E)** rLc1687 for 15 min **(B)**, 30 min **(C)**, 1 h **(D)**, and 2 h **(E)** and then visualized under a light microscope, respectively.

In order to determine the damaging effects of recombinant rLc1687 on *Scuticociliatida*, the ciliates were incubated with the peptide and then observed using a light microscope. As shown in **Figure 9**, in the control group (**Figure 9A**), the ciliates swam freely and maintained *Helianthus annuus-*like morphology. The surface cilia were intact and internal food vacuoles were clearly visible. After incubation for 15 min (**Figure 9B**), the ciliates swam slowly; most of the food vacuole membranes were destroyed. After incubation for 30 min (**Figure 9C**), the food vacuoles completely disappeared and partial cilia disappeared. After incubation for 1 h (**Figure 9D**), the parasites became small and most of the cilia disappeared. After incubation for 2 h (**Figure 9E**), the parasite cell membranes were completely destroyed and intracellular protoplasm spilled out. There is no doubt that rLc1687 has the ability to kill *Scuticociliatida*.

### 3.8 rLc1687 is a relatively stable and safe antimicrobial agent for bony fish

**Figure 10:**
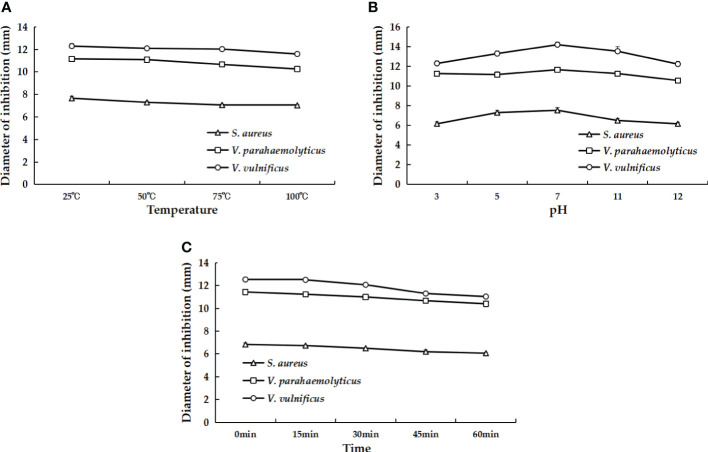
The effects of different temperatures **(A)**, pH values **(B)**, and UV irradiation times **(C)** on the antibacterial activity of rLc1687.

AMPs usually have a certain resistance to various environmental factors. To investigate the possible effects, we set up several different conditions and then measured the antibacterial activity of AMPs. The results indicated that rLc1687 acts as a stable antimicrobial agent against Gram+ and Gram- bacteria at temperatures ranging from 25 to 100 °C, at pH 3-12, and under UV radiation for 15 to 60 min (**Figure 10**).

Hemolytic activity assays were performed on *L. crocea* blood cells. We found that the hemolysis rate of rLc1687 at the extremely high concentration of 1000 mg/L was 2.5% (**Table 1**). According to our results, no significant hemolytic activity was observed against *L. crocea* blood cells. Taken together, it is likely that this peptide is a relatively stable and safe antimicrobial agent for bony fish.

**Table 1:** *Hemolytic activity of* rLc1687 *against* L. crocea blood *cells*.

Treatments	Percentage hemolysis at different concentrations (µg/mL)							
	0	15.625	31.25	62.5	125	250	500	1000
Lc1687	0.00± 0.00	0.003± 0.005	0.005± 0.005	0.008± 0.007	0.017± 0.007	0.014± 0.005	0.018± 0.005	0.025± 0.005
TBS	0.00± 0.00	0.00± 0.00	0.00± 0.00	0.00± 0.00	0.00± 0.00	0.00± 0.00	0.00± 0.00	0.00± 0.00
Ultrapure water	100.00± 0.00	100.00± 0.00	100.00± 0.00	100.00± 0.00	100.00± 0.00	100.00± 0.00	100.00± 0.00	100.00± 0.00

## 4 Discussion

In aquaculture, the accumulation of antibiotics results in the development of resistance among bacterial pathogens. As a result, strong efforts have to be directed toward finding AMPs as alternatives to traditional antibiotics. *B. subtilis* acts as a host cell facilitating soluble and secretory protein expression and is, therefore, particularly effective at producing peptide and protein. In the current study, we screened out a novel antibacterial peptide based on the damaging or killing of the peptide on *B. subtilis* host cells. A drawback of this method is that strong AMPs might not be selected because they kill *B. subtilis* cells too quickly to detect clones, so weakly killed AMPs are preferred. However, *B. subtilis* has a good secretory system, which may decrease the toxicity of strong AMPs. In addition, during the first few hours (12 h), the concentration of strong AMPs is possibly not high enough to kill the cells. These factors make the selection of strong AMPs feasible. In this study, the Lc1687 clone demonstrated a killing effect on *B. subtilis* at 72 h after spotting on the LB plate. Our follow-up investigation confirmed that both repeated selection and extracellular protein exhibit powerful antibacterial activity.

Blast analysis showed that Lc1687 is derived from Ferritin H and located at the C-terminal. Ferritin is a globular protein complex with 24 subunits and has a hollow shell-like structure, which can mineralize up to 4500 iron atoms. In vertebrates, it comprises two Ferritin subunits (heavy subunit-H and light subunit-L) encoded by two different genes (20). Ferritin was involved in the immune response as an acute phase reaction protein in the invasion of pathogens. The transcript level of these ferritins could be significantly induced under infection. Importantly, they were able to exhibit resistant activity to bacterial growth. Zheng et al. verified that a Ferritin in *Scapharca broughtonii* could inhibit the growth of Gram- bacterium *E. coli*, Gram+ bacteria *M. luteus*, and *S. aureus (* 21). Jung et al. demonstrated that Ferritin subunits (H and M) of *Liza haematocheila* possess antibacterial activity against *E. coli* along with iron sequestration activity (22). Ding et al. also indicated that two Ferritin subunits (H and M) from *Megalobrama amblycephala* could inhibit the growth of *Aeromonas hydrophila (* 23). In addition, Ferritin is also involved in the innate defense against viruses. The WSSV copy number in *ferritin*-silenced shrimp was much higher than that in the control group (24). Unfortunately, previous reports have indicated that Ferritin can serve as a bacterial growth inhibitor, whereas no direct killing effect was characterized. In the present study, the extracellular Lc1687 and recombinant rLc1687 exhibited strong bactericidal action against Gram+ bacterium *S. aureus*, Gram- bacteria *V. vulnificus*, and *V. parahaemolyticus*, as well as killing effects on parasitic *Scuticociliatida.* This peptide is considered to be a novel antimicrobial peptide, as no previous reports have revealed that Ferritin fragments are able to constrain the growth of Gram+ or Gram- bacteria.

Lc1687 is a novel anionic antibacterial peptide with α-helical and amphipathic characteristics. Anionic antimicrobial peptides (AAMP) are very rare, and it is thought that these peptides are complements of cationic antimicrobial peptides (CAMP) and have different mechanisms of action. Based on the available experimental evidence, membrane interaction is a key step in the antimicrobial mechanism of AAMPs, including the Shai-Huang-Matsazuki model of membrane interaction and toroidal pore formation, as well as membranolysis *via* tilted peptide formation (25, 26). For example, DCD-1L, one of the best-studied AAMPs, is able to bind to negatively charged bacterial surfaces as an amphiphilic α-helix and then assemble into an oligomeric state. The oligomerization DCD-1L has the capacity to form ion channels in the bacterial membrane, resulting in cell death (27). In addition, α-helical structure is also an important characteristic of AMP. In our study, peptide Lc1687 has the typical α-helical structural characteristics of circular dichroism. The SEM and TEM observations showed that Lc1687 resulted in bacterial cell membrane damage, including shrinkage, distortion, pore formation, and rupture. It appears that some pilis fall off from the surface of *V. vulnificus* and *V. parahaemolyticus.* We incubated this peptide with the genomes of different pathogens, but it did not cause DNA degradation. Based on these investigations, we assume that the possible mechanism of the Lc1687 peptide is to interrupt and rupture the cell membrane, as well as weaken the adhesion and motility of bacteria.

Accumulated evidence has demonstrated that AMPs have antimicrobial activities against a diverse range of pathogens. The Lc1687 peptide was consistent with previously reported AMPs due to its significant inhibitory effect against Gram+ and Gram- bacteria. The MICs for rLc1687 were 31.25-62.5 (5.17-10.33 µM), 15.625-31.25 (2.58-5.17 µM), and 15.625-31.25 (2.58-5.17 µM) µg/mL against *S. aureus*, *V. vulnificus*, and *V. parahaemolyticus*, respectively. Similarly, AAMPs Kappacins A and B, isolated from bovine milk, have been shown to exhibit activity against a range of Gram+ and Gram- oral bacteria with MICs in the 26-44 μM range (28). In contrast, maximin H5 derived from the skin of *Bombina maxima* possesses a net charge of -3 and exhibits weak activity against *S. aureus*, with an MIC of 800 μM and exhibits no activity against Gram- bacteria and fungi (29). In addition to bacterial killing effects, rLc1687 demonstrates forceful anti-parasitic activity against *Scuticociliatida* in our study.

The water environment in which fish live is extremely complex. The production of AMPs and the different forms of administration can cause some effects of the external environment on antibacterial activity. In our study, we found that rLc1687 has a high tolerance to heat, pH, and UV and that it is stable at low pH and high-temperature conditions. Furthermore, AMPs exhibit damaging effects against diverse microorganisms, but their safety in animals and humans is still an open question (30). As previously reported, some AMPs have shown low hemolytic activity against mammalian blood cells (31). In this study, low hemolytic activity in fish was observed at an extremely high peptide concentration. Therefore, we speculated that this peptide, Lc1687, obtained from *L. crocea*, is a relatively stable and safe antimicrobial agent for fish.

In summary, in this study, we screened a novel anionic amphiphilic α-helical peptide, Lc1687, *via* a *B. subtilis* system. Lc1687 is derived from *L. crocea* Ferritin H and exhibits forceful bactericidal and parasiticidal activities, as well as neglectable toxicity to fish. Moreover, Lc1687 has stable antibacterial activity against Gram+ and Gram- bacteria over a wide range of temperatures, pH, and UV radiation time. The functional mechanism of Lc1687 involves destroying the bacterial cell wall and cell membrane. The potent activities endow Lc1687 with promising clinical application prospects against bacterial and parasite infections in fish. Based on these distinctive features, future research will mainly focus on more detailed functional mechanisms and *in-vivo* experiment verification.

## Data availability statement

The datasets presented in this study can be found in online repositories. The names of the repository/repositories and accession number(s) can be found in the article/**Supplementary material**.

## Ethics statement

All experiments were carried out following the principles and protocols of the Animal Care and Use Committee of Fisheries College, Jimei University, China.

## Author contributions

Conceptualization, experimental design, and project administration: DZ and MC; methodology: MC, NL, and XT; supervision: ZW and DZ; writing of the original draft: MC writing—review and editing: XL and DZ. All authors have read and agreed to the published version of the manuscript.
