# Supplementary material for: A novel antimicrobial peptide screened by a Bacillus subtilis expression system, derived from Larimichthys crocea Ferritin H, exerting bactericidal and parasiticidal activities
Source: Front Immunol. 2023 May 18;14:1168517. doi: 10.3389/fimmu.2023.1168517 (PMC10232870; doi:10.3389/fimmu.2023.1168517)
Supplement: Supplementary file 1 [file DataSheet_1.doc]

# Supplementary materials


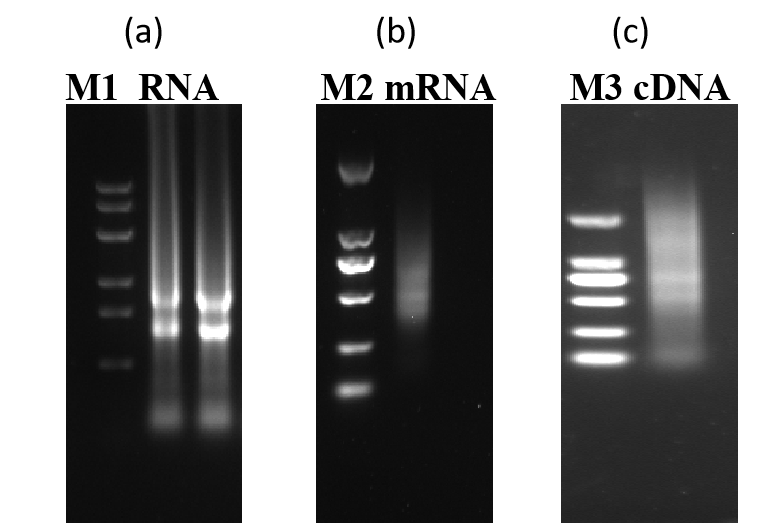


Figure S1 Quality assessment of total RNA, mRNA, and cDNA. (a) Total RNA was extracted using TransZol^TM^ Up Plus RNA Kit (Invitrogen); (b) mRNA was purified from total RNA by PolyATtract® mRNA isolation systems (Promega); (c) Double strand cDNA was synthesized from mRNA using PrimeScript™ double strand cDNA synthesis kit (TaKaRa).


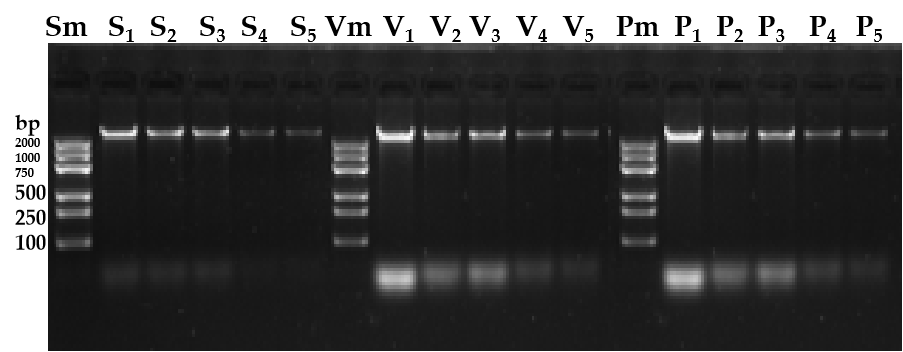


Fig.S2 Gel retardation analysis of rLc1687 binding to bacterial genomic DNA. S/V/Pm: DNA marker; S/V/P_1_: *S. aureus*, *V. vulnificus*, *V. parahaemolyticus* genome DNA. S/V/P_2_-S/V/P_5_: the mixture of different bacterial DNA and rLc1687 with the ratio of 4:1, 3:2, 2:3, and 1:4.

Table S1 Primers used in this investigation

| **Primer** | **Sequence (5'-3')** | **Purpose** |
| --- | --- | --- |
| pBE-S-F | GTTATTTCGAGTCTCTACGG | Confirm the cDNA library quality |
| pBE-S-R | TAACCAAGCCTATGCCTACA |
| Lc1687-F | GCTGATATCGGATCCGAATTCATGTGTGATTTCAT  CGAGACACACT | Expression of rLc1687 |
| Lc1687-R | GTGGTGGTGGTGGTGCTCGAGGCTGCTTTCTTTGCCCAGG |
